# T cell signatures associated with reduced *Chlamydia trachomatis* reinfection in a highly exposed cohort

**DOI:** 10.1172/jci.insight.189388

**Published:** 2025-02-27

**Authors:** Kacy S. Yount, Chi-Jane Chen, Avinash Kollipara, Chuwen Liu, Neha V. Mokashi, Xiaojing Zheng, C. Bruce Bagwell, Taylor B. Poston, Harold C. Wiesenfeld, Sharon L. Hillier, Catherine M. O’Connell, Natalie Stanley, Toni Darville

**Affiliations:** 1 Department of Pediatrics, School of Medicine;; 2 Department of Computer Science; and; 3 Department of Biostatistics, University of North Carolina at Chapel Hill, Chapel Hill, North Carolina, USA.; 4 P.O. Box 247, Topsham, Maine, USA.; 5 University of Pittsburgh School of Medicine and Magee-Womens Research Institute, Pittsburgh, Pennsylvania, USA.; 6 Computational Medicine Program and; 7 Department of Microbiology and Immunology, School of Medicine, University of North Carolina at Chapel Hill, Chapel Hill, North Carolina, USA.

**Keywords:** Immunology, Infectious disease, Bacterial infections, Cellular immune response, T cells

## Abstract

*Chlamydia trachomatis* (CT) is the most common bacterial sexually transmitted infection globally. Understanding natural immunity to CT will inform vaccine design. This study aimed to profile immune cells and associated functional features in CT-infected women and determine immune profiles associated with reduced risk of ascended endometrial CT infection and CT reinfection. PBMCs from CT-exposed women were profiled by mass cytometry, and random forest models identified key features that distinguished outcomes. CT+ participants exhibited higher frequencies of CD4^+^ Th2, Th17, and Th17 double-negative (Th17 DN) CD4^+^ T effector memory (TEM) cells than uninfected participants with decreased expression of T cell activation and differentiation markers. Minimal differences were detected between women with or without endometrial CT infection. Participants who remained follow-up negative (FU–) showed higher frequencies of CD4^+^ T central memory (TCM) Th1, Th17, Th1/17, and Th17 DN but reduced CD4^+^ TEM Th2 cells than FU+ participants. Expression of markers associated with central memory and Th17 lineage was increased on T cell subsets among FU– participants. These data indicate that peripheral T cells exhibit distinct features associated with resistance to CT reinfection. The highly plastic Th17 lineage appears to contribute to protection. Addressing these immune nuances could promote efficacy of CT vaccines.

## Abstract 2

Reduced *Chlamydia trachomatis* reinfection in a highly exposed cohort of women is associated with central Th17 immune memory, highlighting a signature of natural immunity.

## Graphical abstract

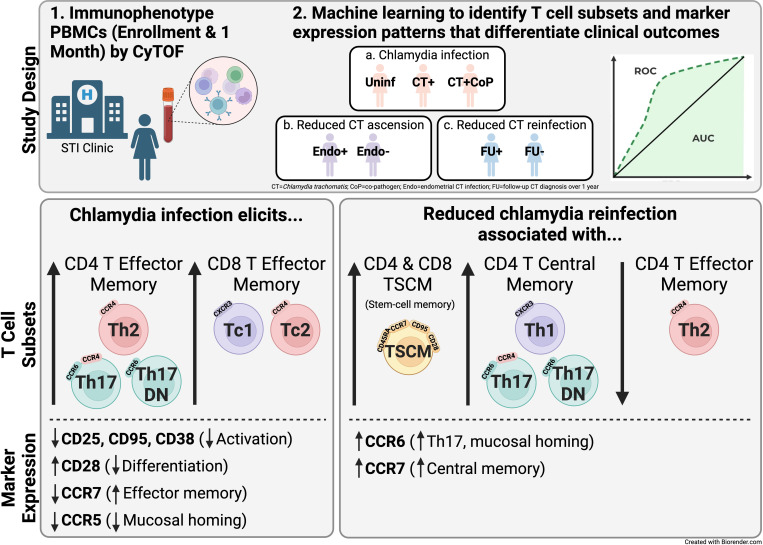

## Introduction

*Chlamydia trachomatis* (CT) is the most common bacterial sexually transmitted infection (STI) globally (1). In women, CT infections can ascend from the cervix to the uterus and oviducts to cause pelvic inflammatory disease (PID) that can result in pelvic pain, infertility, and ectopic pregnancy (2). Although antibiotics effectively treat CT, up to 80% of cases are asymptomatic and may remain undetected (3), contributing to the risk for chronic sequelae (4). Repeated CT infections are common, but they can elicit partially protective immunity, as evidenced by lower reinfection rates and reduced bacterial loads in individuals with prior infections (5, 6). Determining the aspects of natural immunity that drive protection against CT will aid design of an effective vaccine.

Mouse models have revealed the central role of CD4^+^ T cells, particularly IFN-γ–producing Th1 cells, in resolving CT infection and in protection from reinfection, whereas B cells and antibody play secondary roles in protection, and CD8^+^ T cells are largely dispensable (7–15). Th1 cells secrete IFN-γ and TNF-α that promote recruitment and activation of macrophages. IFN-γ also induces expression of indoleamine 2,3-dioxygenase in epithelial cells, which catabolizes tryptophan, an essential nutrient for chlamydia growth (16). A chlamydia*-*specific polyfunctional Th1 clone mediated clearance of murine genital tract infection (17), whereas a Th2 clone was not protective and displayed reduced trafficking to the genital mucosa (18). Additionally, Th2 cells support antibody responses, which have not correlated with protection against CT in humans (19–22). Classical Th17 cells produce IL-17, recruit neutrophils, promote secretory IgA, and are instrumental in protection against many mucosal pathogens but can be pathogenic. Mice lacking T-bet exhibited Th17-skewed immune responses and cleared *C*. *muridarum* infection with similar kinetics as wild-type mice (23). In contrast, mice lacking retinoic acid–related orphan receptor-γt (RORγt; Th17-deficient) showed delayed infection clearance (24), suggesting a potential protective role for Th17 cells. However, in IFN-γ–deficient mice, we observed severe pathology driven by a compensatory increase in Th17 cells and subsequent neutrophil recruitment (25), emphasizing the critical need for balanced Th17 cell responses to avoid immunopathology.

The T Cell Response Against Chlamydia (TRAC) cohort is a potentially unique resource that includes clinical samples and data from women with high exposure to CT and other STIs that was established to enhance understanding of human natural immunity to CT (26). Within this cohort, we observed that peripheral blood CD4^+^ T cells producing CT-specific IFN-γ were associated with protection from reinfection (27), consistent with findings from other studies (28). Additionally, we identified an association of CD8^+^ T cells producing CT*-*specific IFN-γ with protection against endometrial infection (27). In contrast, anti-CT antibodies were found to be insufficient to protect participants from either recurrent or endometrial infection (19, 20). These data indicate that CD4^+^ T cells, and to a lesser extent CD8^+^ T cells, play key roles in immunity against CT reinfection and infection ascension. However, the role of additional T cell subsets and functional markers in mediating resistance to reinfection and endometrial infection remains underexplored.

In this study, we employed high-dimensional immunophenotyping using a cytometry time-of-flight (CyTOF) panel to deeply characterize T cell subsets and functional markers in TRAC participants. The panel was designed to profile Th cell subsets, including T follicular helper (Tfh; CXCR5^+^), regulatory T (Treg; CTLA4^+^CD25^+^CD127^lo^), naive T (TN; CD45RA^+^CCR7^+^), central memory T (TCM; CD45RA^–^CCR7^+^), effector memory T (TEM; CD45RA^–^CCR7^–^), terminally differentiated T effector (TEMRA; CD45RA^+^CCR7^–^), and stem cell–like memory T (TSCM; CD45RA^+^CCR7^+^CD28^+^CD95^+^) cells. TEM and TCM Th cell subsets were defined by the chemokine receptors CCR6, CXCR3, and CCR4, as the presence or absence of these markers is associated with canonical Th1, Th2, and Th17 phenotypes (29–33). Th17 lineage cells exhibit plasticity, adapting chemokine receptor and cytokine expression based on their environment (34, 35). Classical Th17 cells (CCR6^+^CXCR3+CCR4^+^) primarily produce IL-17, supporting neutrophil recruitment and mucosal defense (36). Th1/17 cells (CCR6^+^CXCR3^+^CCR4^–^) combine Th1 and Th17 functions, responding to IL-12 and IL-23 and producing IFN-γ (37). Th17 double-positive (DP) cells (CCR6^+^CXCR3^+^CCR4^+^) produce IFN-γ and low levels of IL-17 (30), while Th17 double-negative (Th 17 DN) cells (CCR6^+^CXCR3^–^CCR4^–^) primarily produce IL-17 and express markers linked to early Th17 cell development, lymph node homing, follicular help, and self-renewal (30, 38).

To deepen our analyses, we incorporated 13 surface markers related to T cell function into a machine learning model to predict clinical outcomes. These surface markers were associated with activation/proliferation (CD71, HLA-DR, CD38, CD25), degranulation/cytotoxicity (CD94, CD107a), differentiation/memory (loss of CD27, CD28, CD127), Treg/checkpoint inhibitor/exhaustion (cytotoxic T lymphocyte antigen 4 [CTLA4], programmed cell death protein 1 [PD-1]), Tfh/germinal center homing (CXCR5), and mucosal migration (CCR5). Analyzing over 400 subset marker pairs required computational methods to handle the high dimensional dataset. Using a random forest model, we evaluated whether T cell subset frequencies and marker expression patterns could distinguish clinical outcomes. When accurate prediction was achieved, feature importance was determined by Gini score (39). Increased frequencies of CD4^+^ TEM Th2 and Th17 lineage subsets, along with reduced expression of T cell activation/differentiation markers in CT+ or CT+ co-pathogen (CT+CoP) participants compared with uninfected individuals, were key to the model’s accurate classification of CT infection. While the model could not reliably predict endometrial infection, it successfully identified features linked to absence of reinfection. The most discriminating features included elevated frequencies of CD4^+^ TCM Th1, Th17, Th1/17, and Th17 DN cells, as well as markers associated with central memory and the Th17 lineage. These data suggest a protective role for Th17 cells, though their contribution to human immunopathogenesis remains unclear.

## Results

### CT genital tract infection elicits monocytes, plasma cells, and CD4^+^ TEM cells in the periphery.

**Figure 1:**
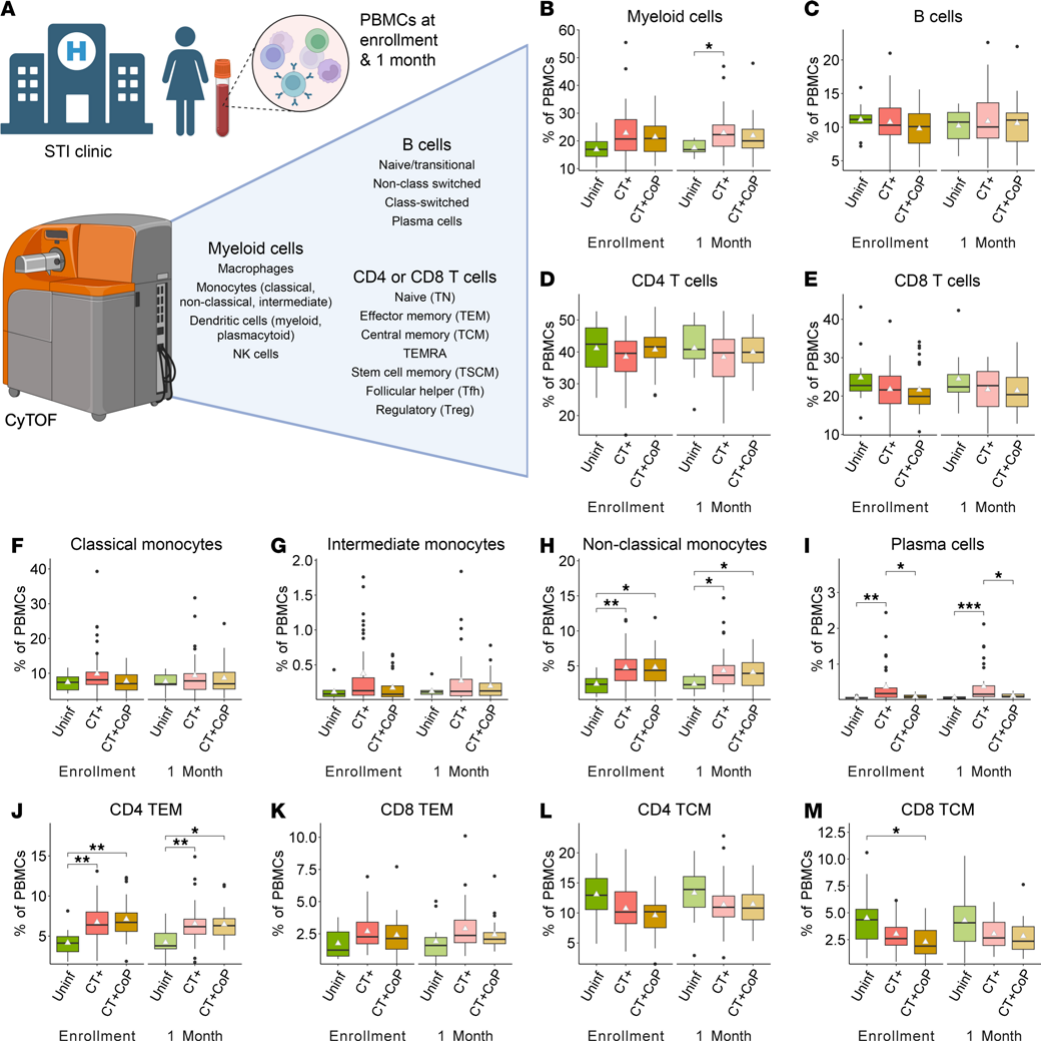
CT genital tract infection elicits monocytes, plasma cells, and CD4^+^ TEM cells. (**A**) Major immune populations defined by manual gating in peripheral blood of 82 participants at enrollment and 1 month after treatment (created with BioRender.com). (**B**–**M**) Population percentages of total PBMCs. White triangle represents mean. Significance determined by Dunn’s test with Bonferroni’s correction for multiple comparisons. Significance symbols used are defined as *P < 0.05, **P < 0.01, ***P < 0.001.

This study acquired CyTOF data from PBMCs of 82 of the 246 participants enrolled in TRAC (26). The sociodemographic characteristics, self-reported STI diagnoses, and exposure histories of this subcohort resemble those previously described for the full cohort (Supplemental Tables 1 and 2; supplemental material available online with this article; https://doi.org/10.1172/jci.insight.189388DS1) (26). Study participants were assigned to infection outcome groups according to the presence of CT or CoPs *Neisseria gonorrhoeae* (NG) and/or *Mycoplasma genitalium* (MG) at enrollment: uninfected (CT^–^NG^–^MG^–^), CT+ (CT^+^NG^–^MG^–^), or CT+CoP (CT^+^; NG^+^ and/or MG^+^). PBMCs, collected at enrollment and 1 month after treatment (1M), were immunophenotyped by CyTOF using a 33-marker panel (Figure 1A and Supplemental Table 3). The manual gating hierarchy and definitions used to determine frequencies of each cell type are outlined in Supplemental Table 4. Frequencies of total myeloid cells, B cells, CD4^+^ T cells, and CD8^+^ T cells were similar across all groups (Figure 1, B–E), as were dendritic cells and NK cells (data not shown). However, classical, intermediate, and nonclassical monocytes were increased in CT+ and CT+CoP women at enrollment and 1M, with significant increases noted in nonclassical monocytes, which are important for antigen presentation (Figure 1, F–H). Macrophage frequencies were variable but slightly increased in CT+ participants at enrollment and 1M (Supplemental Figure 1A). Although frequencies of total B cells were not different at enrollment and 1M, frequencies of plasma cells were increased in CT+ but not CT+CoP participants (Figure 1I). Previous studies have shown that CT drives a strong and specific antibody response (26, 40), consistent with a high frequency of plasma cells. Although frequencies of total T cells were not different, frequencies of CD4^+^ TEM cells were significantly increased in CT+ and CT+CoP participants at enrollment and 1M (Figure 1J), while CD8^+^ TEM cells were only slightly increased in CT+ and CT+CoP participants (Figure 1K). In contrast, CD4^+^ and CD8^+^ TCM cell frequencies were similar or slightly reduced in CT+ and CT+CoP participants compared with uninfected (Figure 1, L and M). Frequencies of CD4^+^ and CD8^+^ TSCM cells were moderately decreased in CT+ and CT+CoP participants at enrollment and 1M, likely a reflection of the proportional increase in TEM cells (Supplemental Figure 1, B and C). Overall, these data demonstrate that CT infection drives increased frequencies of peripheral nonclassical monocytes, plasma cells, and CD4^+^ TEM cells and that responses remain elevated for at least 1 month after treatment.

### CT+ participants have increased peripheral frequencies of Th2 and Th17 lineage CD4^+^ TEM cells.

**Figure 2:**
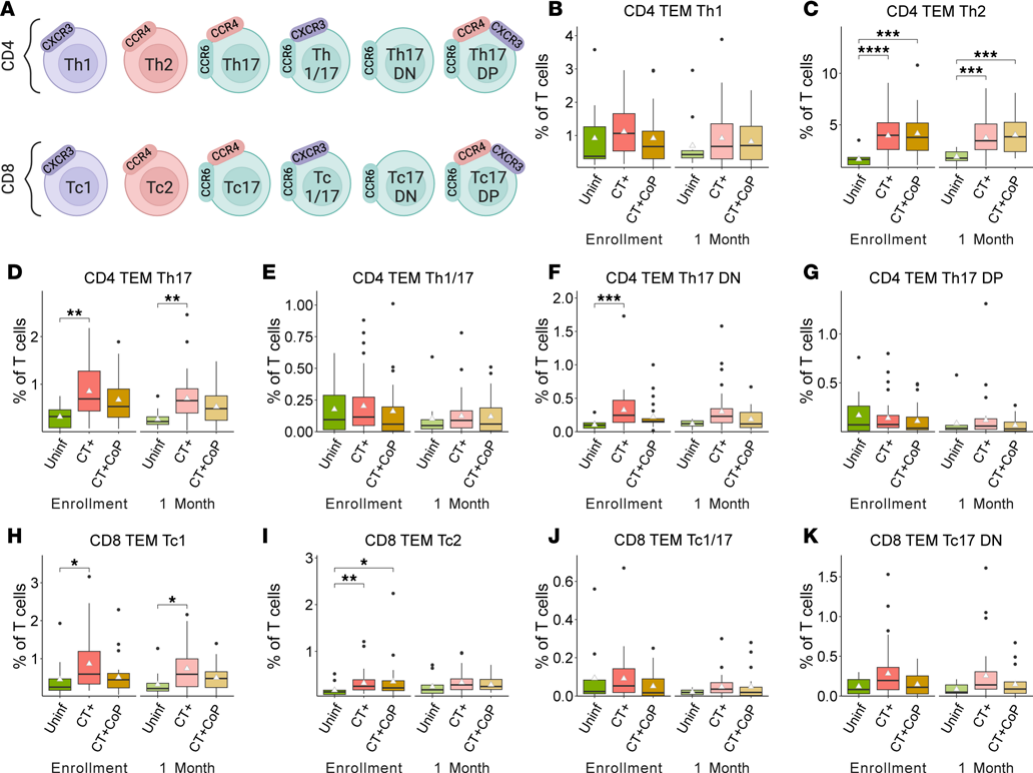
Increased frequencies of Th2 and Th17 lineage CD4^+^ TEM cells among CT+ participants compared with uninfected participants. (**A**) Subsets of CD4^+^ and CD8^+^ TEM cells were identified by expression of chemokine receptors (created with BioRender.com). (**B**–**K**) Population percentages of total T cells. Triangle represents mean. Significance determined by Dunn’s test with Bonferroni’s correction for multiple comparisons. Significance symbols are defined as *P < 0.05, **P < 0.01, ***P < 0.001, ****P < 0.0001.

We used differential expression of the chemokine receptors CCR6, CXCR3, and CCR4 to define Th1, Th2, and Th17 subsets (Figure 2A and Supplemental Table 4) (29, 30, 33). CD4^+^ T cells, and specifically IFN-γ–producing Th1 cells, are broadly accepted as the primary contributors to protection against CT in animals and humans (27, 41), and yet, frequencies of CD4^+^ TEM Th1 cells were not different between CT-infected and uninfected participants (Figure 2B). Instead, frequencies of CD4^+^ TEM Th2 cells were significantly increased in CT+ and CT+CoP participants at enrollment and 1M (Figure 2C), consistent with strong antibody responses detected in response to CT (26, 40). Frequencies of CD4^+^ TEM Th17 and Th17 DN cells were also increased in CT+ participants at enrollment and 1M, whereas there was no difference in frequencies of Th1/17 or Th17 DP cells (Figure 2, D–G). These differences were maintained in CT+CoP participants for CD4^+^ TEM Th2 cells but were dampened in the case of Th17 lineage cells (Figure 2, C–F), suggesting that coinfection dilutes Th17 responses elicited by CT.

We similarly investigated cells comprising the CD8^+^ memory compartment (Tc1, Tc2, Tc17, etc.; Figure 2A and Supplemental Table 4). Frequencies of CD8^+^ TEM Tc1 and Tc2 cells were significantly increased in CT+ participants compared with uninfected (Figure 2, H and I). There was no change in CD8^+^ TEM Tc1/17 cells (Figure 2J), but Tc17 DN cells trended toward an increase in CT+ participants (Figure 2K), both of which paralleled the results for CD4^+^ TEM subsets. Again, these differences were reduced when comparing uninfected and CT+CoP participants. CD8^+^ TEM Tc17 and Tc17 DP cells were not detected because there was very low coexpression of CCR6 and CCR4 on CD8^+^ T cells. CD4^+^ or CD8^+^ TCM subset frequencies were similar or slightly decreased in CT+ participants (data not shown), following the trend previously observed for frequencies of total CD4^+^ and CD8^+^ TCM cells (Figure 1, L and M). Overall, these results suggest that CT infection drives a peripheral adaptive immune response dominated by CD4^+^ and CD8^+^ TEM Th2/Tc2 and CD4^+^ TEM Th17 lineage cells.

**Figure 3:**
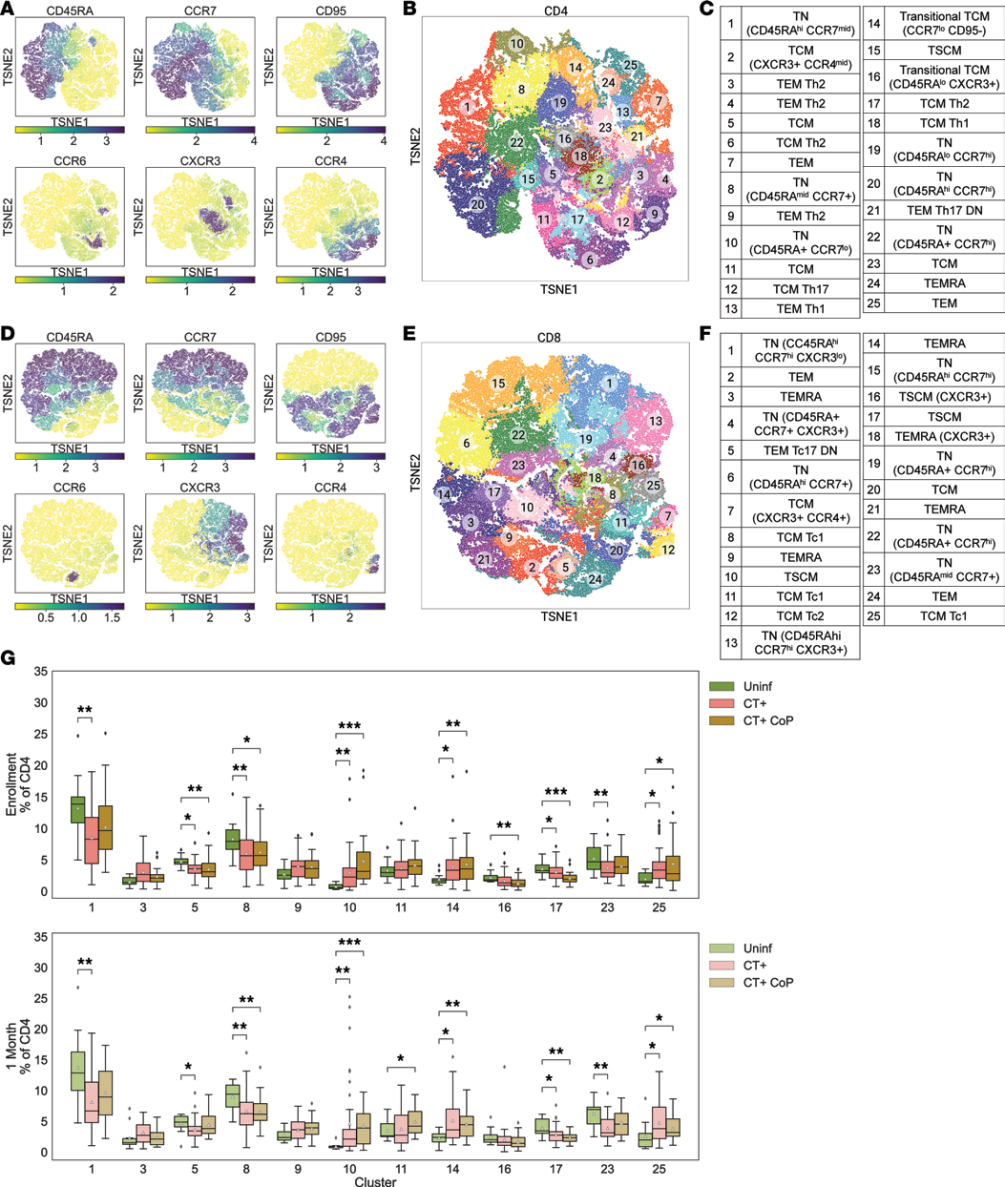
CD4 but not CD8 automated gating frequency features differentiate CT+ and CT+CoP participants from uninfected participants. (**A**–**F**) Automated gating by *k*-means clustering and cluster annotation. (**A** and **B**) Expression of phenotypic markers (CD45RA, CCR7, CD95, CCR6, CXCR3, and CCR4) was used to partition CD4^+^ T cells into 25 *k*-means clusters. (**C**) Resulting CD4^+^ clusters were annotated based on marker expression (see Supplemental Tables 4 and 5). (**D** and **E**) CD8^+^ T cells were partitioned into 25 *k*-means clusters based on the expression of the same phenotypic markers as for CD4^+^ T cells and (**F**) were annotated. (**G**) Box plots representing frequencies of CD4^+^ clusters with significant differences in frequency (Wilcoxon *P* < 0.05). Statistical comparisons between CT+ or CT+CoP and uninfected by Wilcoxon test. Significance symbols are defined as *P < 0.05, **P < 0.01, ***P < 0.001. White triangle represents mean. TSNE, t-distributed stochastic neighbor embedding.

We then implemented an unsupervised clustering-based strategy to increase the resolution of our findings obtained by supervised manual gating (Figure 3, A–F). Clustering with the *k-*means algorithm partitioned cells based on expression of 6 phenotypic markers (CD45RA, CCR7, CD95, CCR6, CXCR3, CCR4). We previously defined TSCM cells as CD45RA^+^CCR7^+^CD28^+^CD95^+^ by manual gating (Supplemental Table 4), but CD28 had a relatively small range of expression and was not strongly discriminatory across clusters. Therefore, this marker was excluded for unsupervised discrimination of T cell subsets, effectively simplifying the TSCM definition to CD45RA^+^CCR7^+^CD95^+^. Following clustering, memory and Th subtypes were annotated based on marker expression as done previously (Supplemental Tables 4 and 5, and Figure 3, A–F). Annotation revealed similar clusters to manual gating overall, but enhanced resolution allowed detection of heterogeneity in TN subsets (Figure 3, C and F). CD4^+^ clusters 1, 20, and 22 and CD8^+^ clusters 6, 15, and 22 expressed high levels of both CD45RA and CCR7, representing the classical definition of TN cells. However, CD4^+^ clusters 10, 14, 16, and 19 expressed lower levels of CD45RA or CCR7, suggesting that these cells were differentiating from a naive state to a memory state. Additionally, CD8^+^ clusters 1, 19, 4, and 13 were CD45RA^+^CCR7^+^ like traditional TN cells but also expressed CXCR3, a marker typically reserved for memory Th1 cells. Studies have shown that naive human CD8^+^ T cells expressing CXCR3 have greater potential for effector differentiation (42).

Clusters with frequencies that were statistically significant (*P* < 0.05 by Wilcoxon test) between uninfected and CT+ or CT+CoP participants at enrollment or 1M are represented in Figure 3G as a distribution of cluster frequencies per participant. Frequencies of CD4^+^ clusters 10 (TN with low CCR7), 14 (transitional TCM), 25 (TEM), and 3 and 9 (TEM Th2) were increased in CT+ and CT+CoP participants compared with uninfected, while frequencies of clusters 1 and 8 (classical TN), 5 and 23 (TCM), and 17 (TCM Th2) were decreased, suggesting that CD4^+^ T cells respond to CT by evolving away from a naive state. Similar immune signatures were observed at enrollment and 1M, suggesting that changes in immune responses in the periphery are maintained after treatment. These findings were consistent with manual gating, where overall CD4^+^ TEM cells (Figure 1J), and especially TEM Th2 cells (Figure 2C), were increased in CT-infected participants compared with uninfected while overall frequencies of CD4^+^ TCM cells were trending toward decrease (Figure 1L).

### CT+ and CT+CoP participants express reduced T cell activation and differentiation markers on several CD4^+^ and CD8^+^ subsets compared with uninfected participants.

Although the unsupervised clustering approach lent confidence to our previous findings and uncovered previously unappreciated nuance in the TN population, it demonstrated limited resolution of low-frequency T cell subsets defined by manual gating, such as Th17 lineage cells, that were of particular interest to us. Therefore, for further analysis of marker expression on each T cell subset, we used manually gated subsets. We implemented a random forest machine learning model trained by T cell subset frequencies as well as expression levels of the phenotypic markers used above to define T cell subsets (CD45RA, CCR7, CD95, CCR6, CXCR3, CCR4) and 13 surface markers that relate to T cell function to predict clinical outcomes. The panel included surface markers associated with the following T cell functions: activation/proliferation (CD71, HLA-DR, CD38, CD25), degranulation/cytotoxicity (CD94, CD107a), differentiation/memory (loss of CD27, CD28, CD127), Treg/checkpoint inhibitor/exhaustion (CTLA4, PD-1), Tfh/germinal center homing (CXCR5), and mucosal migration (CCR5). The upper quartile expression of each of these 19 markers was calculated for each CD4^+^ or CD8^+^ manually gated subset (as previously defined, Figure 2 and Supplemental Table 4) and for each participant. These upper quartile expression levels (functional features) were then used in addition to the manually gated frequency features to train the random forest model. This machine learning approach allowed interrogation of over 400 features defined by subset marker pairs.

**Figure 4:**
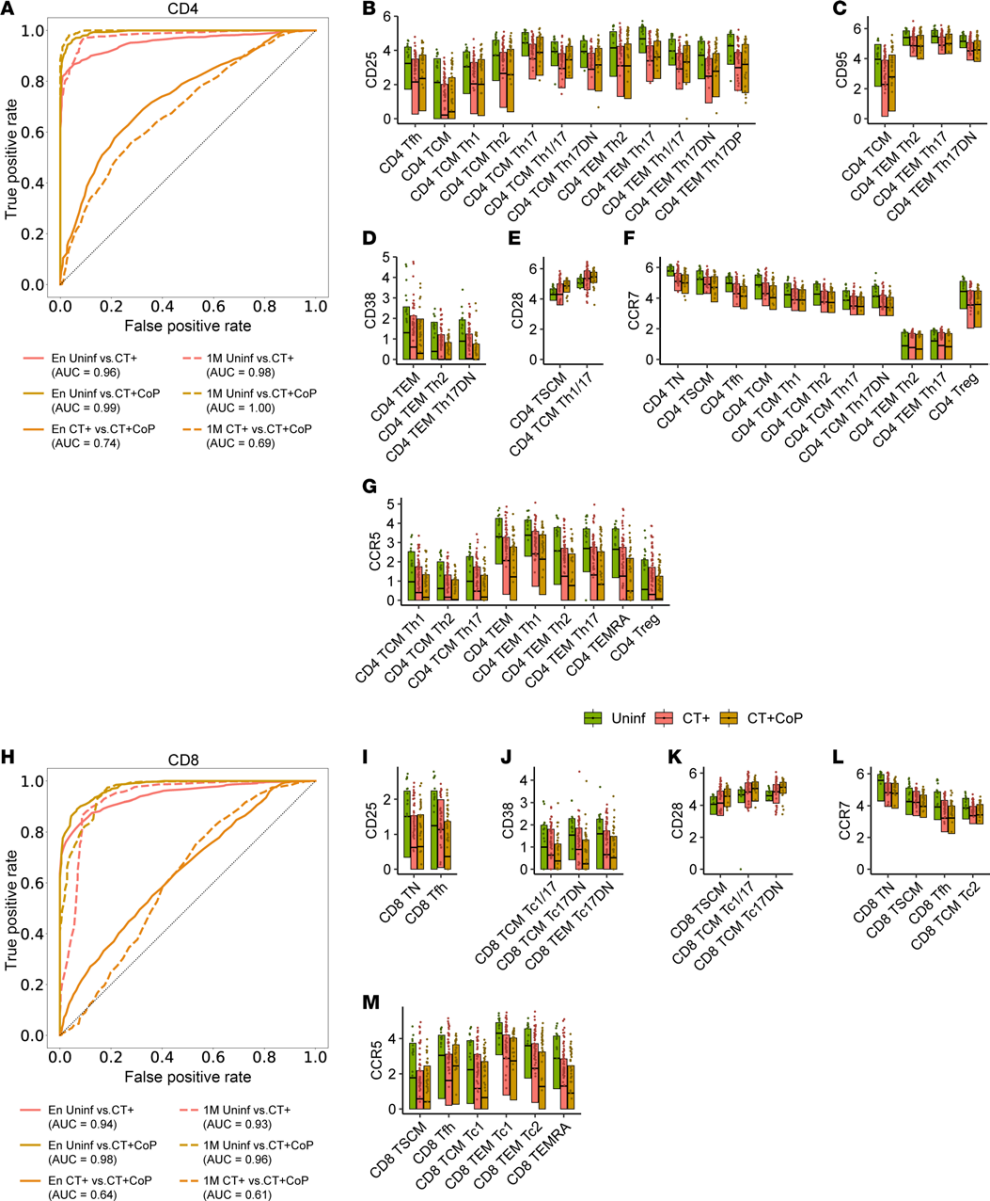
CT+ and CT+CoP participants express reduced T cell activation and differentiation markers on several CD4^+^ and CD8^+^ subsets compared with uninfected participants. A random forest model trained on (**A**) CD4^+^ or (**H**) CD8^+^ manually gated subset frequency features and upper quartile expression features was used to discriminate between uninfected, CT+, and CT+CoP outcomes at enrollment (En) or 1 month (1M). Receiver operating characteristic (ROC) curves describe performance of the model. Top informative markers of (**B**–**G**) CD4^+^ or (**I**–**M**) CD8^+^ T cells were identified by feature importance analysis at En (see Supplemental Tables 6 and 7 for additional features and 1M analysis). Features were included on the plot if they were statistically significant (as defined by *P* < 0.001 by Wilcoxon rank sum test between outcomes) for at least 1 comparison between uninfected versus CT+ or uninfected versus CT+CoP. Box plots represent the overall distribution of marker expression. Individual points represent the upper quartile expression for each participant in the group. AUC, area under the ROC curve.

Classification of uninfected versus CT+ or CT+CoP participants’ subset frequency and functional features was successful for CD4^+^ (Figure 4A; AUC range 0.96–1.00) or CD8^+^ T cells (Figure 4H; AUC range 0.93–0.98). Together, these results suggest that frequency and functional features of CD4^+^ and CD8^+^ T cells contribute to distinction between CT-infected participants and uninfected participants. The similarity between model success at enrollment and 1M suggests that these signatures were maintained after infection resolution.

To identify prominent cellular subsets that contributed to prediction, a measure of feature importance, or Gini score, was assigned to each frequency feature or functional feature (39). Briefly, the Gini score encodes the extent to which each frequency feature or functional feature provides the model with useful information, such that subsets with high Gini scores are likely to be the most useful in distinguishing between clinical outcomes. The Gini scores for predicting classification of infection status are represented in Supplemental Table 6 for CD4 features and Supplemental Table 7 for CD8 features. In general, features contributing to accurate classification of uninfected versus CT+ and uninfected versus CT+CoP were similar for the models informed by CD4 and CD8 features, consistent with similar accuracy between the 2 comparisons and weak classification accuracy for CT+ versus CT+CoP. A subset of upper quartile functional features with the highest importance by Gini score and significant difference in expression between uninfected and CT+ or CT+CoP by Wilcoxon test (*P* < 0.001) is shown in Figure 4, B–G (CD4) and I–M (CD8). Several subsets of both CD4^+^ and CD8^+^ T cells in CT-infected participants expressed lower levels of CD25 (IL-2Rα), CD95 (Fas), and CD38, markers associated with T cell activation and antigen experience, than the same subsets in uninfected participants (Figure 4, B–D, I, and J). Loss of costimulatory molecules CD27 and CD28 is associated with T cell differentiation, and CD28 expression was higher in some CD4^+^ and CD8^+^ subsets from CT+ and CT+CoP participants than in uninfected participants (Figure 4, E and K), suggesting that peripheral blood from infected participants contained less differentiated T cells. Activated, antigen-experienced, differentiated T cells may have trafficked to the infected genital tract, resulting in a reciprocal decrease in differentiation markers in the blood. Several subsets, especially TSCM and TCM subsets, also expressed lower levels of CCR7 in CT+ or CT+CoP participants than uninfected participants (Figure 4, F and L). This aligns with increased frequencies of TEM (CCR7^–^) cells in CT+ and CT+CoP participants and suggests that the TCM cells of infected participants are shifted more toward an effector phenotype. Additionally, expression of the mucosal homing marker CCR5, a marker known to be important for trafficking of T cells to the genital tract during murine chlamydial infection (43, 44), was decreased in several subsets in CT+ or CT+CoP participants compared with uninfected participants (Figure 4, G and M). This CCR5 expression pattern could suggest that CCR5^+^ T cells had migrated to the genital mucosa at the time of sampling. Frequencies of T cell subsets, especially TEM Th2, TEM Th17 DN, and TEM Th17 among CD4^+^ T cells (Supplemental Table 6) and TEM Tc2 and generic TEM among CD8^+^ T cells (Supplemental Table 7), were also important contributors to the differentiation of CT+ from uninfected participants. Trends in feature importance analysis were similar for both CD4^+^ and CD8^+^ T cells at 1M (Figure 4 and Supplemental Tables 6 and 7), suggesting maintenance of the T cell signature following treatment and infection resolution.

Discrimination of CT+ versus CT+CoP participants using CD4 or CD8 frequency demonstrated only weak to moderate success, with greater success from CD4 features (Figure 4A; AUC range: 0.69–0.74) than CD8 (Figure 4H; AUC range: 0.61–0.64). The top contributing feature by Gini score was higher CTLA4 expression on CD4^+^ T cell subsets among CT+CoP participants compared with CT+ participants (Supplemental Table 6). CTLA4 plays an important role in CD4^+^ Treg function by downregulating T cell activation. NG evades host immunity by inducing TGF-β, which is important for Treg development. The increase in CTLA4 expression on CD4^+^ T cell subsets among CT+CoP participants compared with CT+ participants could be reflective of NG-mediated immune evasion.

### Neither T cell subset frequencies nor functional expression features predict endometrial CT infection.

Endometrial biopsies from all participants were tested for CT, NG, and MG infection at enrollment, enabling bisection of the CT+ and CT+CoP groups into CT infections that had ascended to the upper genital tract (Endo+) and CT infections that were limited to the cervix (Endo–). All major cell populations and T cell subsets were defined by manual gating (Supplemental Table 4), and comparisons of population frequencies were performed between Endo+ and Endo– participants. Endo+ participants exhibited significantly increased frequencies of CD4^+^ TSCM cells, which were not observed in Endo+ CT+CoP participants (Supplemental Figure 2A). Frequencies of CD8^+^ TEM cells were not different between Endo+ and Endo– outcomes for CT+ or CT+CoP participants (Supplemental Figure 2B). Among Endo– participants, there was a trend toward increased frequencies of CD8^+^ TEM Tc17 and TCM Tc17 DN cells (Supplemental Figure 2, C and D), suggesting expansion of these cells may contribute to protection from ascending CT infection. However, the random forest model trained on manually gated subset frequencies with the addition of upper quartile expression of functional markers (Supplemental Figure 2, E and F) failed to accurately classify Endo+ from Endo– participants based on CD4 or CD8 features. Overall, there were relatively minimal differences in the frequency and functional marker expression patterns in peripheral blood T cell subsets between participants with or without ascended CT infection.

### Increased TSCM, CD4^+^ TCM Th1, and CD4^+^ TCM Th17 lineage subsets but decreased CD4^+^ TEM Th2 cells in peripheral blood are associated with absence of CT reinfection.

Individuals treated for CT often experience repeat infections (6). Nevertheless, evidence exists for partially protective immunity after infection (28). TRAC study participants were followed for 1 year to determine if they became reinfected (26). We again bisected CT+ and CT+CoP groups into FU+ (positive for CT by diagnostic test or self-report at any follow-up visit) and FU– (completed at least 3 out of 4 follow-up visits, and CT negative by diagnostic test and self-report at all visits). Five out of 20 participants in the CT+ FU+ group tested positive for CT at 1M, while none of the participants in the CT+CoP FU+ group tested positive for CT at 1M. Removing these 5 participants in the CT+ FU+ group from the analysis did not change the overall differences observed between FU– and FU+ participants.

**Figure 5:**
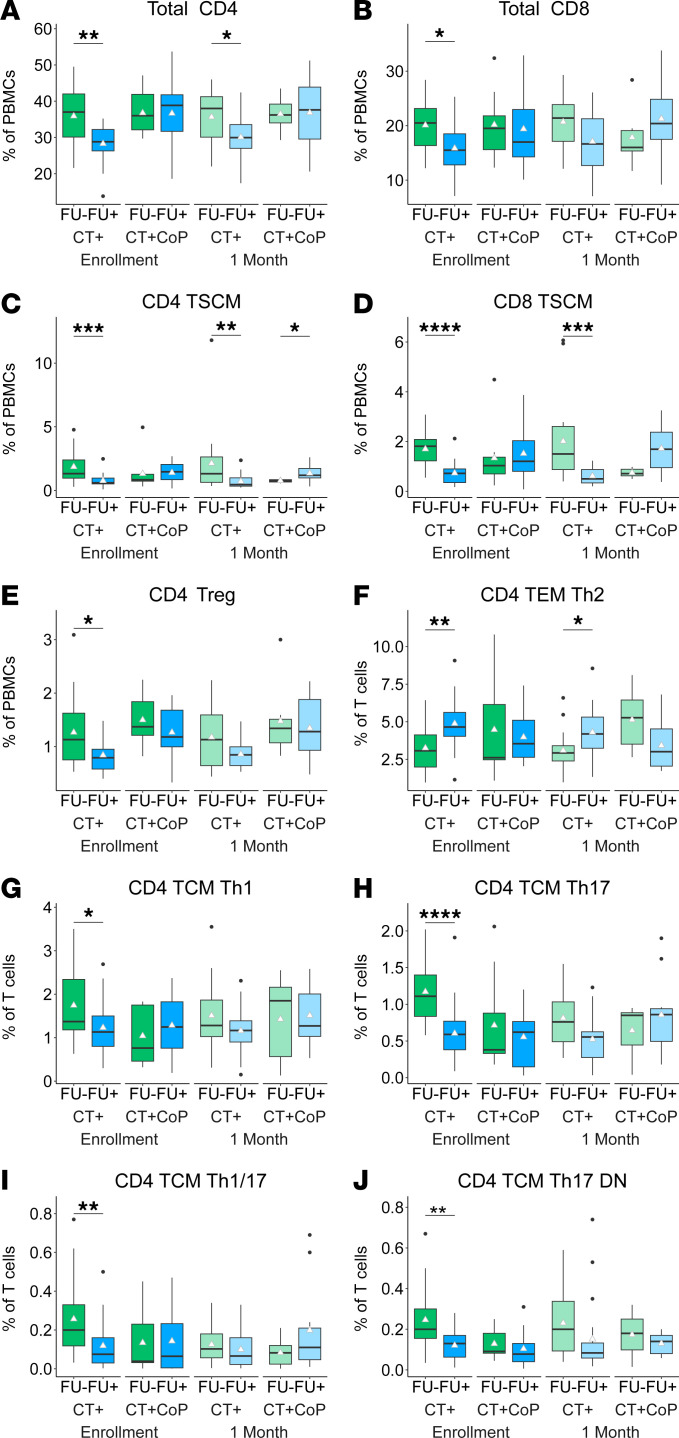
CD4^+^ TCM Th1 and Th17 family subsets are enriched in peripheral blood of participants without documented CT reinfection. Frequencies of manually gated T cell subsets were stratified by follow-up negative (FU–) and follow-up positive (FU+) status. (**A**–**J**) T cell subset percentages. White triangle represents mean. Statistical significance determined by Wilcoxon rank sum test. Significance symbols are defined as *P < 0.05, **P < 0.01, ***P < 0.001, ****P < 0.0001.

We compared frequencies of manually gated T cell subsets between FU– and FU+ individuals. Frequencies of CD4^+^ and CD8^+^ total T cells and CD4^+^ and CD8^+^ TSCM cells were enriched in FU– participants without coinfection at enrollment and remained expanded 1 month after treatment (Figure 5, A–D). CD4^+^ Tregs were enriched in FU– participants at the enrollment visit (Figure 5E). Interestingly, frequencies of CD4^+^ TEM Th2 cells were increased in FU+ participants (Figure 5F), suggesting that though these cells are induced by CT infection (Figure 2C), they are not protective. In contrast, frequencies of CD4^+^ TCM Th1, Th17, Th1/17, and Th17 DN cells were increased in FU– participants (Figure 5, G–J). These data suggest CD4^+^ and CD8^+^ TSCM and CD4^+^ TCM Th1/Th17 lineage cells are important for natural immunity against incident CT infection. Differences in frequencies of Th17 family subsets between FU– and FU+ participants were detected among only CT+ and not CT+CoP participants, which suggests that Th17 immune responses specific to CT are diluted by coinfection.

We then repeated the comparison using frequencies of clusters generated by *k*-means (Figure 3, A–F) and observed trends toward higher frequencies of clusters 15 (TSCM) and 12 and 21 (Th17 family) and lower frequencies of clusters 3, 4, and 9 (Th2 TEM) in FU– participants compared with FU+ (Supplemental Figure 3; see Figure 3C for cluster annotations). These trends were consistent with results obtained by traditional manual gating (Figure 5) and suggest a protective role for TSCM and TCM Th17 lineage cells but not for Th2 TEM cells. However, there was limited resolution of Th17 family subsets by clustering compared with manual gating.

### Central memory and Th17 lineage markers distinguish participants who did not become reinfected with CT.

**Figure 6:**
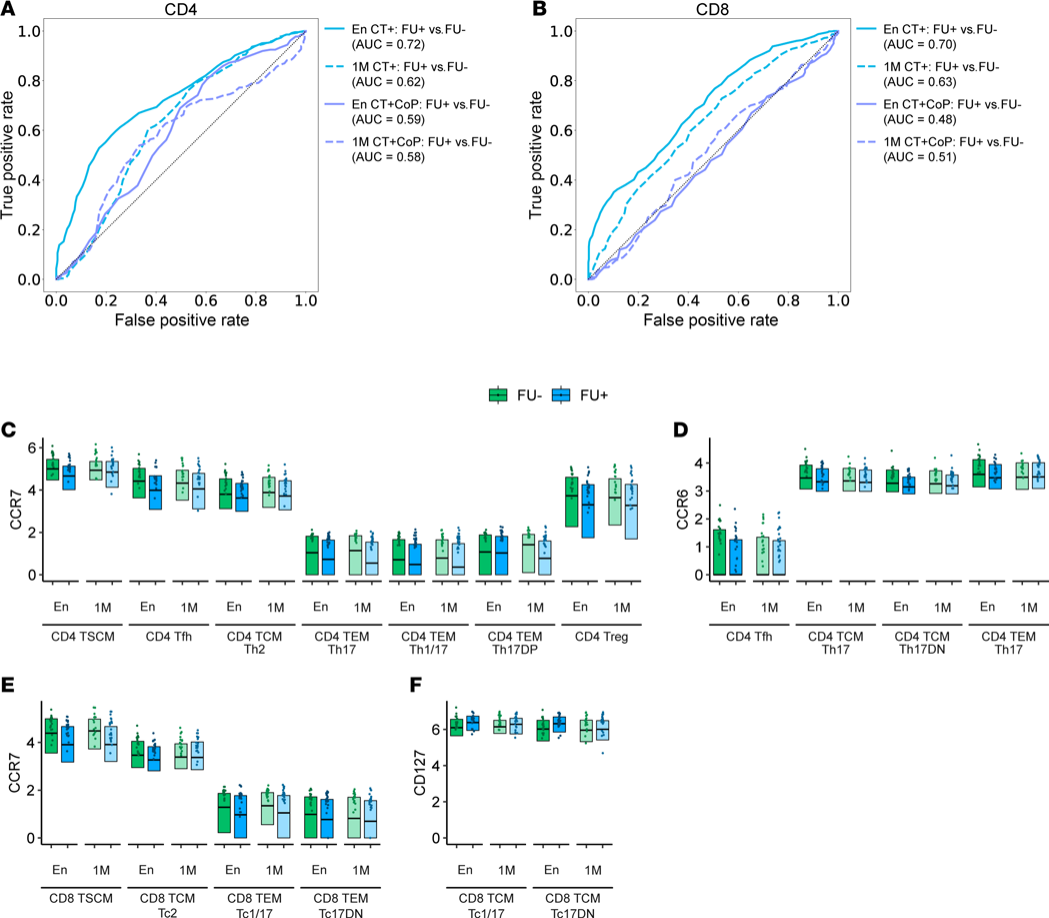
Increased central memory and Th17 lineage markers distinguish participants without documented CT reinfection. A random forest model trained on (**A**) CD4^+^ or (**B**) CD8^+^ manually gated subset frequency features and upper quartile expression features was used to discriminate between FU+ and FU– groups at En or 1M. ROC curves visualize the performance of the model. Top markers of (**C** and **D**) CD4^+^ or (**E** and **F**) CD8^+^ T cells among CT+ participants were identified by feature importance analysis (see Supplemental Table 8). Features were included on the plot based on a significance threshold of *P* < 0.05 by Wilcoxon rank sum test for FU+ versus FU– at enrollment. Box plots represent the overall distribution of marker expression. Individual points represent the upper quartile expression for each participant in the group.

We used a random forest machine learning model trained with manually gated T cell subset frequencies and functional expression level features to classify FU+ versus FU– status (Figure 6). Classification of FU+ versus FU– was most accurate for CT+ participants at enrollment (CD4 ROC AUC = 0.72; CD8 ROC AUC = 0.70) (Figure 6, A and B). Analysis of feature importances by Gini score identified cell types and subset-specific expression patterns that were most distinct (Supplemental Table 8). Among CT+ participants, the most prominent CD4 features contributing to successful FU– versus FU+ classification were decreased frequencies of Th2 cells and increased frequencies of Th17 family subsets in FU– participants (Supplemental Table 8). The upper quartile functional features with highest importance by Gini score and significant difference in expression between FU+ and FU– by Wilcoxon test (*P* < 0.05) are highlighted in Figure 6, C–F. Important CD4 features included increased expression of CCR7 on T cell subsets among FU– participants, consistent with increased central memory, and increased CCR6 expression on Th17 subsets among FU– participants, consistent with increased Th17 lineage (Figure 6, C and D). Important CD8 features among FU– participants included increased frequencies of CD8^+^ TSCM cells, increased expression of CCR7 (similar to the CD4 model), and reduced expression of CD127 (IL-7 receptor, loss of which is associated with differentiation and memory) (Supplemental Table 8 and Figure 6, E and F). Overall, the model was most successful in distinguishing FU+ from FU– participants among CT+ participants when informed by CD4^+^ T cell frequencies at enrollment. CD8^+^ T cell features at 1M were less informative, and coinfection with NG or MG diluted the success of the model. Overall, these results suggest a protective role for CD4^+^ TCM Th17 lineage cells against CT reinfection.

## Discussion

Using samples from a well-characterized and clinically relevant cohort, this study identified immune signatures associated with reduced incidence of CT reinfection through high dimensional immunophenotyping of PBMCs. We found that increased frequencies of specific T cell subsets, particularly CD4^+^ TCM, Th1, and Th17 lineage cells, correlated with reduced reinfection. These findings highlight key components of natural immunity to CT that could inform vaccine development.

Broad immune cell profiles in peripheral blood differed between uninfected and CT+ participants but were similar between those with CT monoinfection and coinfection with NG or MG. CT+ participants had increased frequencies of nonclassical (CD14^–^CD16^+^) monocytes, which play roles in antigen presentation, T cell proliferation, tissue surveillance, and Fc receptor–mediated phagocytosis (45). Plasma cells were elevated in CT monoinfection, consistent with strong but nonprotective antibody responses observed during human CT infection (19–22), but remained similar to uninfected levels in coinfected (CT+CoP) participants. This may result from NG opacity proteins binding carcinoembryonic antigen-related cellular adhesion molecule 1 (CEACAM1 or CD66a) on B cells, triggering inhibitory signals that promote B cell death (46). Since plasma cells also express CD66a (47), this interaction could further reduce plasma cell frequencies.

CD4^+^ TEM frequencies were significantly higher (*P* < 0.01, Figure 1J) in CT+ and CT+CoP than uninfected participants, while CD8^+^ TEM frequencies were not significantly different between groups. This aligns with previous findings in TRAC participants, where CD4^+^ T cells showed higher IFN-γ production and broader CT antigen recognition compared with CD8^+^ T cells (27). Lower CD8^+^ TEM frequencies may reflect PD-1 receptor engagement on CD8^+^ T cells, inhibiting generation of CT-specific memory CD8^+^ T cells (48).

Investigation of CD4^+^ TEM subsets revealed that CT+ participants had higher frequencies of peripheral Th2, Th17, and Th17 DN cells, while Th1 cells remained unchanged compared with uninfected participants. CD4^+^ Th1 cells are classically defined by IFN-γ production and CXCR3 or CCR5 expression (29) and are critical for chlamydial clearance and resistance to reinfection in mouse models (17, 43, 44) and humans (27, 28). The lack of Th1 expansion in peripheral blood may explain the high rates of CT reinfection. However, Th1 cells express CXCR3 and CCR5, which bind the chemokines CXCL10 and CCL5 (RANTES), respectively (49). We previously reported detecting these chemokines in cervical secretions of TRAC participants (50), and they are highly expressed in genital tract tissues of *Chlamydia* *muridarum*–infected mice (43). Since we found decreased expression of CCR5 on T cell subsets among CT+ and CT+CoP participants, it is possible that despite overall expansion of Th1 cells, they were preferentially recruited to the site of infection and consequently not increased in the periphery. In contrast, CD4^+^ Th2 cells were significantly enriched in CT+ and CT+CoP participants, aligning with high antibody titers to CT infection that are insufficient to prevent reinfection (19–22). Th17 cells are defined primarily by CCR6 expression (51) and exhibit high plasticity, gaining or losing chemokine receptors and effector functions, such as cytokine production (34). While Th17 cells can be pathogenic, they can also contribute to protection against mucosal pathogens (52, 53). Frequencies of classical Th17 and Th17 DN cells were elevated in CT+ participants compared with uninfected individuals but to a lesser extent in cases of coinfection. Parallel CD8^+^ TEM subsets, including Tc2 and Tc17 DN cells, were also increased in CT+ and CT+CoP participants, though to a lesser extent than CD4^+^ counterparts.

An important goal of the study was to analyze surface markers associated with T cell function, including activation/proliferation (CD71, HLA-DR, CD38, CD25), degranulation/cytotoxicity (CD94, CD107a), differentiation/memory (loss of CD27, CD28, CD127), Treg/checkpoint inhibitor/exhaustion (CTLA4, PD-1), Tfh/germinal center homing (CXCR5), and mucosal migration (CCR5). We examined over 400 subset marker combinations using a random forest model to determine whether T cell subset frequencies and functional expression features were distinct between observed clinical outcomes of CT infection (uninfected, CT+, CT+CoP), ascension (Endo+/–), or reinfection (FU+/–).

The model successfully classified CT+ or CT+CoP participants from uninfected participants using CD4 or CD8 features. Increased frequencies of CD4^+^ TEM Th2, Th17, and Th17 DN cells and distinct expression patterns of CD25, CD95, CD38, CCR7, CD28, and CCR5 were key features. CD25 (IL-2 receptor), crucial for T cell activation and proliferation; CD95 (Fas), involved in apoptosis but also a marker of antigen experience and stem cell memory; and CD38, a receptor involved in activation, adhesion, and signaling, were all expressed at lower levels in CT+ and CT+CoP participants compared with uninfected, indicating reduced activation and differentiation. Higher expression of CD28, a costimulatory molecule downregulated upon T cell differentiation, among CT+ participants also suggested reduced differentiation. Lower CCR7 expression on T cells among CT+ and CT+CoP participants correlated with higher TEM frequencies. Reduced CCR5 expression, critical for effector T cell trafficking to the genital tract (44), was also observed. The correlation of these marker expression patterns with CT infection indicates that although CD4^+^ TEM cells were increased in peripheral blood of CT+ participants, they were not more activated or differentiated compared with uninfected individuals. The chronicity of chlamydial infections may suppress peripheral T cell differentiation and promote the migration of activated, differentiated T cells from the peripheral blood to the infected mucosa. Supporting this, prior studies reported higher CD38 and CCR5 expression on T cells in the genital tract compared with peripheral blood in CT+ women (54, 55), and another study found that CT-specific IFN-γ responses in PBMCs were correlated with those in endometrial biopsies of highly exposed female sex workers (56). These findings underscore the complex dynamics between peripheral and mucosal immune responses during chlamydial infection.

Since CT ascension is a prerequisite for upper tract disease, we analyzed T cell subsets associated with ascended infection. Peripheral immune cell profiles were similar between participants with endometrial infection (Endo+) and those with infection limited to the cervix (Endo–). CD8^+^ TEM Tc17 DN cells trended higher in Endo– participants, consistent with previous findings that CT-specific CD8^+^ T cells weakly associated with reduced CT ascension (27). CD4^+^ TSCM frequencies were significantly increased in Endo+ (*P* < 0.05 for Endo+, Supplemental Figure 2A) and FU– (*P* < 0.001 for FU–, Figure 5C) participants, possibly reflecting a robust memory response driven by higher bacterial burden in endometrial infection (26). However, a random forest model failed to distinguish Endo+ from Endo– infections using peripheral immune features, suggesting peripheral immune responses are insufficient for identifying ascended CT infection. Future studies will focus on immune cell populations in cervical and endometrial TRAC samples to better understand local immunity against CT ascension.

Several T cell subsets were strongly associated with absence of recurrent CT infection. FU– participants showed sustained increases in total CD4^+^ T cells and higher frequencies of CD4^+^ and CD8^+^ TSCM cells. TSCM cells resemble TN cells in their expression of CD45RA and CCR7, but they also express CD95, a marker of antigen experience and memory (57). These cells have a high propensity for stemness, self-renewal, and multipotency (57). These features may explain their association with FU– status.

Higher TEM Th2 cell frequencies were associated with FU+ outcome, while increased TCM Th1, Th17, Th1/17, and Th17 DN cells were associated with FU– status. *K*-means cluster analysis verified these patterns, with TSCM and Th17 lineage clusters enriched in FU– participants and TEM Th2 clusters reduced, underscoring the importance of memory and lineage plasticity in immunity to CT. These findings suggest that Th1 and Th17 lineage cells play critical roles in protection from CT reinfection. A recent study detected enrichment of *Mycobacterium tuberculosis*–specific Th17-like cells in people who were resistant to *Mycobacterium tuberculosis* infection following exposure compared with those who were latently infected (58). The increased stemness and differentiation potential of TSCM and Th17 lineage cells (32, 59), especially Th17 DN cells (30, 38), may allow them to adapt quickly to fight infection.

Our finding that TCM subsets (TCM Th1, TCM Th17, TCM Th1/17, and TCM Th17 DN cells) rather than TEM subsets were associated with FU– status is consistent with our detection of CT antigen–specific CD4^+^ TCM cells persisting months after treatment in TRAC participants (60). Similarly, *Chlamydia* *pneumoniae*–specific CD4^+^ TCM cells were enriched over TEM cells in healthy seropositive donors (61). These results suggest that TCM cells play a critical role in long-term protection against reinfection, even though active CT infection primarily drives TEM cell expansion.

A random forest model distinguishing FU– from FU+ participants identified key differences, including increased frequencies of TCM Th17, Th17 DN, Th1/17, and Th1 cells and decreased TEM Th2 cell frequencies, in FU– participants. Higher expression of CCR7, a marker of TCM cells and essential for lymph node trafficking, was observed on several subsets among FU– participants, emphasizing the critical role of TCM cells for protection against reinfection. Expression of CCR6 was also higher on several T cell subsets among FU– participants. In addition to being a canonical marker of Th17 cells, CCR6 binds CCL20 expressed in mucosal tissues and facilitates migration to the site of infection. High CCR6 expression is also associated with increased secretion of Th17 effector cytokines and chemokines (IL-17A, IL-17F, IL-22, and CCL20) (59), further emphasizing its role in mucosal immunity and reinfection resistance.

The finding that CD4^+^ Th1 and Th17 subsets were associated with reduced reinfection highlights their importance in vaccine development. Different vaccine platforms and adjuvants can shape Th responses (62). Vaccines using CAF01 (63), or a STING agonist (64), successfully induced Th1 and Th17 responses in mice, reducing chlamydial burden upon challenge. In contrast, a viral vector vaccine failed to elicit antigen-specific CD4^+^ T cell responses or protection (65), underscoring the need for platforms that effectively target these Th subsets.

Our study has some limitations. While we identified immune cell populations increased in CT+ participants, we did not restimulate cells with CT antigens to verify their specificity. However, direct immunophenotyping allowed for confident identification of T cell subsets without the risk of receptor downregulation. A separate study of the same cohort found low-frequency sustained CT-specific CD4^+^ IFN-γ responses (60). Nonetheless, an important open question is whether Th17 and Th17 DN cells produce CT-specific IL-17 or IFN-γ, and this will be addressed in future studies. Another limitation was the use of frozen PBMC samples, potentially impacting the proportions of cell subsets that would have been observed in fresh samples. However, using frozen samples allowed identical processing conditions for all samples and facilitated the conservation of both samples and resources. Moreover, it enabled balanced selection of samples based on the longitudinal analysis of repeated infections across at least 3 follow-up visits, spanning a period of 8 to 12 months or more.

Overall, we found enrichment of CD4^+^ TEM subsets, specifically Th2, Th17, and Th17 DN cells, in CT+ individuals, while CD4^+^ TCM Th1, Th1/Th17, and Th17 cells were associated with resistance to reinfection. These findings advance our understanding of natural immunity to CT and guide vaccine development strategies.

## Methods

### Sex as a biological variable

The TRAC cohort was restricted to people with a cervix and uterus because an objective of the study was to determine factors associated with ascension of CT from the cervix to the endometrium.

### Study population

This study involved a subset of 82 of the 246 participants enrolled in the TRAC cohort (26). TRAC participants were asymptomatic and presented to the clinic for STI screening because of sexual behavior that put them at risk for CT (16). None had signs or symptoms of PID. At enrollment, all participants received antibiotics recommended by the Centers for Disease Control and Prevention for treatment of CT and NG, and individual participants who tested positive for STI at any follow-up visit were retreated (16).

### Definition of clinical classifications

#### Uninfected, CT+, and CT+CoP groups.

Nucleic acid amplification testing (NAAT) was used to test cervical swabs collected from study participants at enrollment for CT, NG, and MG. Participants whose swabs tested negative for all 3 STIs at enrollment were classified as “uninfected.” Participants testing positive for CT but negative for NG and MG were classified as “CT+.” Enrollees testing positive for CT and NG, MG, or both were classified as “CT+CoP.” Of 82 total TRAC participants studied, 12 were uninfected, 44 were CT+, and 26 were CT+CoP (Supplemental Table 9). Of 26 CT+CoP participants, 11 were CT^+^NG^+^MG^–^, 12 were CT^+^MG^+^NG^–^, and 3 were CT^+^NG^+^MG^+^.

#### Endometrial positive and endometrial negative CT.

Endometrial biopsies were also tested by NAAT to determine whether infection was limited to the cervix or had ascended to the upper genital tract. Within CT+ and CT+CoP groups, enrollees testing positive for CT at the cervix but not the endometrial biopsy were classified as Endo–, while those testing positive for CT at both sites were classified as Endo+. No participants tested positive for CT solely in the endometrial sample. Of 44 CT+ participants, 19 were Endo+ and 25 were Endo–. Of 26 CT+CoP participants, 11 were Endo+ and 15 were Endo– (Supplemental Table 9).

#### Follow-up CT positive and follow-up CT negative.

NAAT testing was performed on cervical brush samples at enrollment and at 1-, 4-, 8-, and 12-month follow-up visits. Participants with a positive cervical CT test at enrollment and a positive cervical CT test or self-reported CT diagnosis from an outside clinic in the time since last visit at any follow-up visit (regardless of how many visits they completed) were classified as FU+. Participants who tested positive for cervical CT at enrollment, completed at least 3 out of 4 follow-up visits, tested negative for cervical CT at all follow-up visits, and self-reported no CT diagnosis from an outside clinic in the time since last visit at any follow-up visit were classified as FU–. A total of 9 participants attended fewer than 3 follow-up visits (FU N/A) and were excluded from follow-up analysis. Of CT+ participants, 19 were FU–, 21 were FU+, and 4 were FU N/A. Of CT+CoP participants, 9 were FU–, 12 were FU+, and 5 were FU N/A (Supplemental Table 9). All uninfected samples were FU–, and the group was therefore not bisected.

### PBMC CyTOF

PBMCs were isolated from whole blood via a density gradient using lymphocyte separation media (Corning). Cells were frozen in 90% FBS with 10% DMSO and stored in liquid nitrogen until thawed for analysis. Dead cells were stained with 1 μM Cell-ID Cisplatin 198-Pt (Fluidigm). Cells were stained with an antibody cocktail comprising 33 antibodies (Supplemental Table 3) and human Fc block (eBioscience) in cell staining buffer (Fluidigm), followed by staining with secondary antibody (anti–FITC-144Nd, custom antibody conjugated by the University of North Carolina [UNC] Mass Cytometry Core). Stained cells were fixed with 2% paraformaldehyde in PBS followed by 1:2,000 Cell-ID Intercalator-Ir-125 μM (Fluidigm) in fix and perm buffer (Fluidigm). Cell concentrations were adjusted to 0.5 × 10^6^ cells/mL in Cell Acquisition Solution (Fluidigm) with Four Element Calibration beads (Fluidigm). Samples were acquired on a Helios instrument (Fluidigm) by the UNC Mass Cytometry Core.

### Data analysis

We defined T cell subsets by manual or automated gating. Raw CyTOF files were cleaned using the Pathsetter algorithm (66).

#### Manual gating.

Cleaned FCS files were analyzed in Cytobank (Beckman Coulter) and FlowJo (BD Biosciences) software. The manual gating strategy and surface marker designations selected for immune cell phenotypes are summarized in Supplemental Table 4. Some gated CD8^+^ populations were undetectable (<0.1% of T cells) and were excluded from further analysis (Supplemental Table 4).

#### Automated gating.

Batch effects were removed from the dataset using the combat algorithm (67). A random subset of 15,000 cells was selected from each sample, resulting in 1,230,000 total cells for further analysis. We then performed automated cell population discovery followed by feature engineering, as outlined in Stanley et al. (68). CD4^+^ and CD8^+^ T cell populations were each partitioned into 25 clusters using *k*-means clustering. Briefly, each cell was represented by expression of 6 phenotypic markers (CD45RA, CCR7, CD95, CCR6, CXCR3, CCR4), which were input to the clustering algorithm. Moreover, each cell was labeled in terms of 1 of the 25 clusters, based on its expression across these 6 phenotypic markers (Supplemental Table 5). Modeled after canonical immune features that are often computed based on manual gates, we engineered per-cluster frequency features, where for each sample, we computed the proportion of its cells assigned across each of the 25 clusters.

#### Feature engineering.

Whereas previous studies have created functional features based on the mean or median expression of functional markers in each cluster (68, 69), some functional markers in this dataset were expressed by a small proportion of cells, such that there were some cases where median expression of a marker was 0. Therefore, we computed the upper quartile expression of each marker across each manually gated T cell subset for each sample. For samples with 0.1% events assigned to a particular subset, the upper quartile features were imputed with the mean of each subset marker feature. Additionally, subset marker features for which upper quartile expression was below 2 (range 0–500) were excluded from the training dataset. Upper quartile features were arcsinh-transformed with cofactor 5. In all, 19 frequency features and 233 upper quartile expression features were included in the CD4 model training dataset, and 14 frequency features and 174 upper quartile expression features were included in the CD8 model training dataset.

#### Model training.

We trained random forest models using engineered manually gated frequency features and upper quartile expression features to classify and predict clinical outcomes for each participant. For each clinical outcome comparison, we performed 30 trials of 5-fold crossvalidation, whereby each fold used 80% of samples for training and 20% for testing. Reported AUCs represent the mean accuracy across 30 trials. For biological interpretability of each of the trained random forest models, we further computed per-feature importance measures with the Gini index (39). A higher per-feature Gini score implied that the feature was important in the clinical prediction task. We further imposed directionality to the computed per-feature Gini scores to readily identify condition-specific, predictive features by first choosing a particular clinical outcome to be the positive (negative) direction, such that we multiplied the Gini score by 1 (–1), if it was associated with the positive (negative) direction.

### Statistics

Statistical calculations were computed in R and python. Statistical tests used are outlined in the appropriate figure legends. *P* < 0.05 was used to determine a significant result. For all box-and-whisker plots, the box itself extends from the first quartile (Q1) to the third quartile (Q3). A line within the box represents the median value. Whiskers extend to the most extreme data point within 1.5 × IQR from Q1 and Q3. White triangle represents mean.

### Study approval

The institutional review boards for human research of the University of Pittsburgh and the UNC approved the study protocol. All participants provided written informed consent at the time of enrollment and agreed to be contacted to return for follow-up visits 1, 4, 8, and 12 months after enrollment.

### Data availability

CyTOF data (including antibody sources and catalog numbers) are available for download via ImmPort at https://www.immport.org under study accession number SDY2772. Manually gated T cell subset frequencies reported in the figures are available in Supporting Data Values. Code for (i) automated gating, (ii) feature engineering, (iii) random forest training and Gini feature importance analysis, and (iv) upper quartile expression box plots are available via GitHub at https://github.com/ksyount/TRAC_PBMC_CyTOF (commit ID f401cfa).

## Author contributions

KSY, AK, XZ, NS, and TD designed research studies. KSY and AK conducted experiments. HCW and SLH collected participant samples and performed diagnostic testing. TBP advised regarding mass cytometry panel design. KSY, CJC, CL, NVM, and CBB analyzed data. KSY, CJC, NS, CMO, and TD wrote the manuscript. All authors reviewed the manuscript.

## Supplementary Material

Supplemental data

Supplemental tables 1-9

Supporting data values

## Version 1 02/27/2025

In-Press Preview

## Version 2 04/08/2025

Electronic publication
