# Supplementary material for: T cell signatures associated with reduced Chlamydia trachomatis reinfection in a highly exposed cohort
Source: JCI Insight. 2025 Feb 27;10(7):e189388. doi: 10.1172/jci.insight.189388 (PMC11991011; doi:10.1172/jci.insight.189388)
Supplement: Supplemental data [file jciinsight-10-189388-s007.pdf]

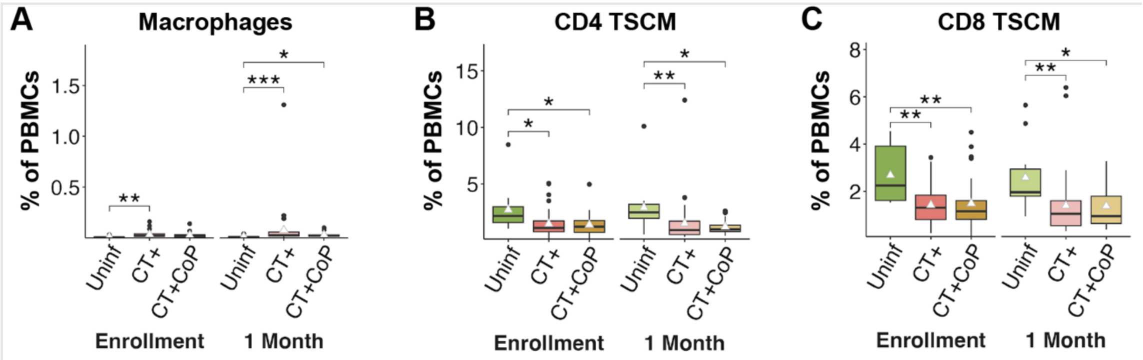

Supplemental figure 1. Frequencies of manually gated cell subsets among participants. Population percentages of total PBMCs. White triangle represents mean. Significance determined by Dunn's test with Bonferroni correction for multiple comparisons. (Uninf = CT- and NG- and MG-; CT+ = CT+ and NG- and MG-; CT+CoP [Co-Pathogen] = CT+ and NG+ and/or MG+)

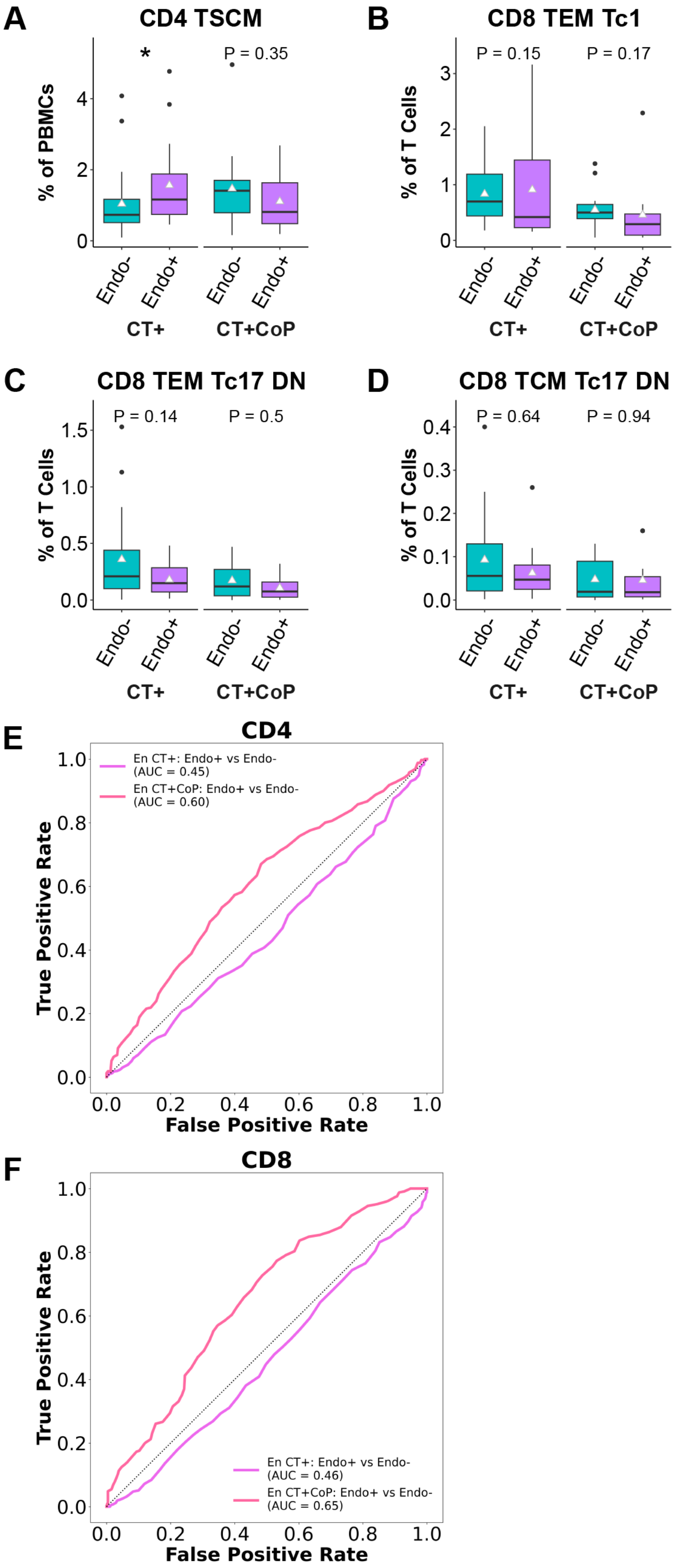

Supplemental figure 2. Features of CD4 and CD8 T cells are not distinct between CT+ participants with or without infection that ascended to the endometrium.
Frequencies of manually gated T cell subpopulations were stratified by cervix only CT (Endo-) or cervix and endometrial CT (Endo+) status. (A-D) T cell subset percentages. White triangle represents mean. Significance determined by Wilcoxon rank-sum test. A random forest model trained on (E) CD4 or (F) CD8 manually gated subset frequency features and upper quartile expression features was used to discriminate between Endo+ and Endo- groups at enrollment (En). Receiver operating characteristic (ROC) curves visualize the performance of the model. (AUC = area under the ROC curve).

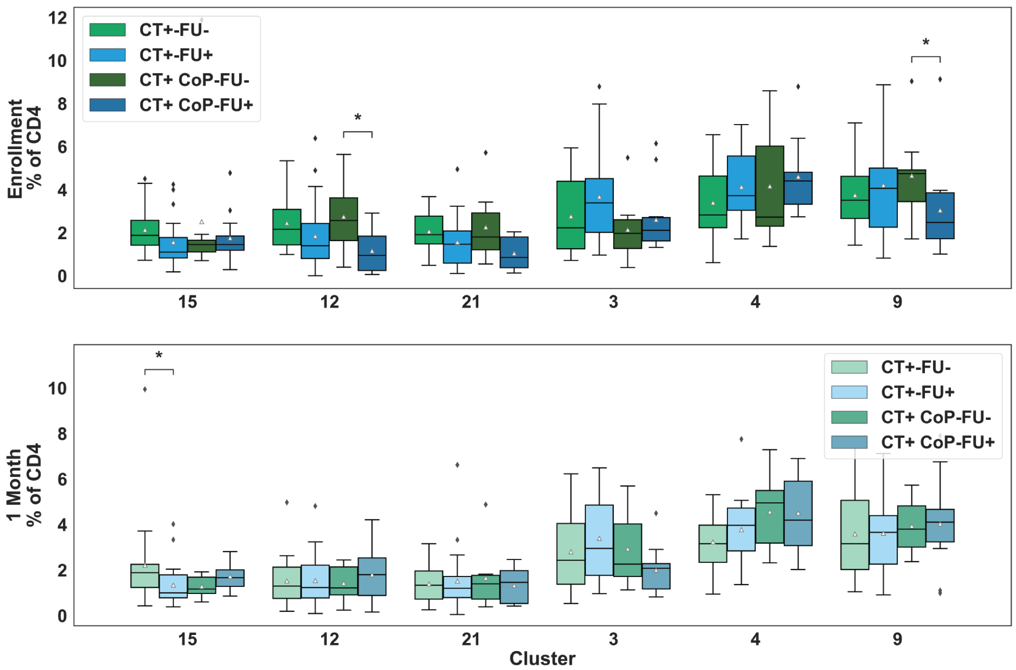

Supplemental figure 3. Differences in CD4 T cell subset frequencies between FU- and FU+ participants by automated gating are similar to those observed by manual gating.
Boxplots representing frequencies of select CD4 clusters (reference Fig 3A-C) among participants. Statistical comparisons between FU- and FU+ by Wilcoxon test. White triangle represents mean.
